# Association Study of a Serotonin Receptor 2A Gene -1438A/G Polymorphism and Anxiety-Related Traits

**DOI:** 10.4306/pi.2008.5.4.244

**Published:** 2008-12-31

**Authors:** Kye-Hyun Kim, Hee-Yeon Woo, Se-Won Lim

**Affiliations:** 1 Department of Obstetrics Gynecology, Kangbuk Samsung Hospital, Sungkyunkwan University School of Medicine, Seoul, Korea.; 2 Department of Laboratory Medicine, Kangbuk Samsung Hospital, Sungkyunkwan University School of Medicine, Seoul, Korea.; 3 Department of Psychiatry, Kangbuk Samsung Hospital, Sungkyunkwan University School of Medicine, Seoul, Korea.

**Keywords:** Anxiety, Anxiety sensitivity, Polymorphism, Serotonin 2A receptor

## Abstract

**Objective:**

The aim of this study was to investigate the relationship between the -1438A/G polymorphism of serotonin receptor 2A (5HTR2A) and anxiety-related traits in Korean adolescent females.

**Methods:**

A total of 174 Korean adolescent females were tested for the -1438A/G polymorphism of 5HTR2A using polymerase chain reaction (PCR)-based methods. Anxiety-related traits were evaluated using the Anxiety Sensitivity Index (ASI) and the trait form of the Spielberg State-Trait Anxiety Inventory (T-STAI).

**Results:**

There was no difference between the genotypes with respect to scores pertaining to anxiety-related traits. Although the G allele carriers (GG or AG genotype) scored lower on the psychological subscale of the ASI (4.76±3.00 vs 5.98±4.00, p=0.038), this difference was not significant after Bonferroni correction.

**Conclusion:**

These findings suggest that the -1438A/G polymorphism of 5HTR2A might not be associated with anxiety sensitivity or trait anxiety.

## Introduction

The 5-hydroxytryptamine (serotonin) receptor 2A (5HTR2A) gene has been implicated as a functional candidate gene for various psychiatric illnesses, especially mood^1^,^2^ and anxiety disorders.^3^,^4^ However, it is very difficult to determine the involvement of a specific gene in mood and anxiety disorders due to the high rate of comorbidity between these two disease categories. Therefore, a recent genetic association study^5^ focused on anxiety-related traits associated with susceptibility to anxiety disorders because these traits have a higher heritability than the transient disease status.^6^

It is generally accepted that anxiety disorders have both genetic and environmental causes. However, the influence of the environment is hard to evaluate, and it often serves as a confounding factor in traditional genetic association studies. Lusher et al.^7^ proposed that variability in subject age may lead to inconsistent results in genetic association studies of personality traits.

Therefore, it can be assumed that the influence of environmental factors on the development of anxiety disorders will be lower in younger subjects. The purpose of the present study was to investigate the relationship between the -1438A/G polymorphism of 5HTR2A and anxiety-related traits in a homogenous group of Korean adolescent females (i.e., similar age, residence, and high school of attendance).

## Methods

### Subjects

The Ethics Committee of Kangbuk Samsung Hospital approved this study. A total of 204 Korean adolescent females aged 16 or 17 years (mean±SD; 16.73±0.70 years) were invited to participate in this study. Informed consent was obtained from all subjects and their parents. All of the volunteers were recruited from two neighboring high schools for women in Seoul. Clinical interviews were performed to exclude subjects with major medical or psychiatric conditions. All subjects were asked to complete the validated Korean version of the Beck Depression Inventory (BDI),^8^ the trait form of the Spielberg State-Trait Anxiety Inventory (T-STAI),^9^ and the Anxiety Sensitivity Index (ASI).^10^ The physical, psychological, and social concern subscales of the ASI^11^ were also evaluated, as suggested by McWillams and Cox.

Only subjects with BDI scores of <21 were tested for the -1438A/G polymorphism of 5HTR2A because a previous study reported that the -1438A/G polymorphism is associated with depressive disorders in the Korean population.^1^ The final study sample was comprised of 174 unrelated Korean adolescent females.

### Genotyping

Approximately 5-10 mL of venous blood was collected in an EDTA tube. Genomic DNA was extracted from leukocytes using a QIAamp Blood Kit (Qiagen, Germany). The -1438A/G polymorphism was examined according to the method described by Chee et al.^12^ Polymerase chain reaction (PCR) was performed with the following primers: sense, 5'-CTGGGTGGCATATTTCTGCT-3' and antisense, 5'-ACCAAGGGACTCCTGGTTTC-3'. The amplification mixture contained 3 µL of DNA, 5 µL of 10 PCR buffer, 4 µL of 2.4 mM deoxynucleoside triphosphate (dNTP), 1 µL of each primer, 35.75 µL of distilled water, and 0.25 µL of Taq polymerase. Samples were amplified using a thermocycler with 35 cycles of 5 minutes (min) at 94℃, 1 min at 60℃, and 1 min at 72℃. After a final cycle of 9 min at 72℃, the amplified DNA was digested with the restriction endonuclease MspI, which cuts the DNA at the -1438G site. The product was electrophoresed in 2% agarose gels and stained with ethidium bromide.

### Statistical analysis

The presence of Hardy-Weinberg equilibrium for genotype frequencies was calculated using a χ^2^ test. Genotype differences in continuous variables were evaluated by a t-test or one-way analysis of variance (ANOVA). All analyses were performed using SPSS for Windows (SPSS, Chicago, IL, USA), with the level of statistical significance set at p<0.05. Bonferroni correction for multiple testing was performed because we analyzed 3 subscales of the Anxiety Sensitivity Index.

## Results

The genotype frequencies were as follows: AA (n=43), 24.7%; AG (n=89), 51.1%; and GG (n=42), 24.1%. The frequencies of the A and G alleles were 50.3% and 49.7%, respectively, which are similar to the frequencies previously reported in Korean^1^ and Japanese^14^ populations. The genotype distribution was in Hardy-Weinberg equilibrium (χ^2^=0.09, df=1, p=0.761). One-way ANOVA did not show significant differences between the genotype groups with respect to the T-STAI, total ASI, and three ASI subscale scores. The G allele carriers (GG+AG genotypes) scored lower than the non-carriers (AA genotype) on the ASI-psychological subscale (AA genotype, 5.98±4.00; GG and AG genotypes, 4.76±3.00; t=2.094, p=0.038). However, this difference was not significant after Bonferroni correction for multiple testing (p=0.152). There were no significant differences between the two groups with respect to the other subscales of the ASI (i.e., ASI-physical and ASI-social), total ASI, and T-STAI (Table 1).

## Discussion

To our knowledge, this is the first report of an association between the -1438A/G polymorphism of 5HTR2A and anxiety-related traits. Although Pearson et al.^15^ reported that the presence of the A allele was associated with a more significant increase in promoter activity than the G allele, the functional significance of the -1438A/G polymorphism of 5HTR2A remains unclear, as the previous study results were inconsistent.^16^ Although a previous study reported that medications that target 5HTR2A and have serotonin reuptake inhibitor (SSRI) properties modulate anxiety^17^ and a clinically-proven positive therapeutic effect of SSRIs for the treatment of anxiety disorders suggested a possible association between anxiety-related traits and the -1438A/G polymorphism of 5HTR2A, our study showed negative results.

In this study, carriers of the G allele (GG and AG genotypes) scored lower on the ASI-psychological subscale than non-carriers. However, this difference was insignificant after Bonferroni correction for multiple testing.

Our negative findings may be related to the inherent characteristics of anxiety, which is a clinically and etiologically heterogeneous disorder with a wide range of symptoms. Another explanation for our result may be a lack of sensitivity in the scales used in this study. However, considering that the previous association study, which also used the T-STAI, reported an association between the Val66Met polymorphism of the brain-derived neurotrophic factor (BDNF) gene and trait anxiey,^5^ it is less likely that our negative finding is due to the limited sensitivity of the scales used.

This study has several limitations. First, although we used the BDI to exclude subjects in a severe state of depression, a structured clinical interview was not performed, and the information used in this study was obtained via a self-report questionnaire. Second, only female subjects were included in this study. Considering that there are gender differences in anxiety sensitivity,^18^ our results cannot be generalized to males. The final limitation, which may prove to be a major weakness of this study, is its lack of enough statistical power. The sample size of this study may be too small to allow for the detection of a relevant effect of a single gene on a complex phenotype. Although this study has several limitations, our study population was very homogenous, and thus the number of confounding factors was minimized.

In conclusion, the results of the present study suggest that the -1438A/G polymorphism of 5HTR2A might not be associated with anxiety-related traits, such as anxiety sensitivity and trait anxiety, in Korean adolescent females. Further investigations with larger sample sizes are warranted in order to confirm our findings.

## Figures and Tables

**TABLE 1:**
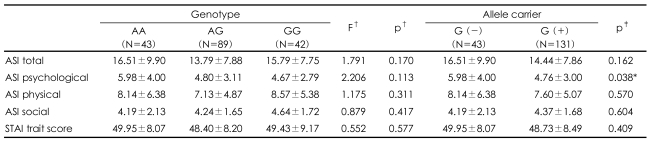
Comparison of anxiety-related traits according to the -1438A/G polymorphism of 5HTR2A and G allele carrier state

^*^Not significant after Bonferroni correction p=0.128, ^†^One-way ANOVA, ^‡^Independent t-test. ASI: Anxiety Sensitivity Index, STAI: Spielberg State-Trait Anxiety Inventory, G (+): allele subjects include GG or AG genotype, G (-): allele subjects include AA genotype only
